# Chemometric Analysis of a Ternary Mixture of Caffeine, Quinic Acid, and Nicotinic Acid by Terahertz Spectroscopy

**DOI:** 10.1021/acsomega.2c03808

**Published:** 2022-09-27

**Authors:** Phatham Loahavilai, Sopanant Datta, Kiattiwut Prasertsuk, Rungroj Jintamethasawat, Patharakorn Rattanawan, Jia Yi Chia, Cherdsak Kingkan, Chayut Thanapirom, Taweetham Limpanuparb

**Affiliations:** † National Electronics and Computer Technology Center, 112 Thailand Science Park, Pathum Thani 12120, Thailand; ‡ Department of Engineering Physics, Tsinghua University, Beijing 100084, China; § Mahidol University International College, Mahidol University, Nakhon Pathom 73170, Thailand

## Abstract

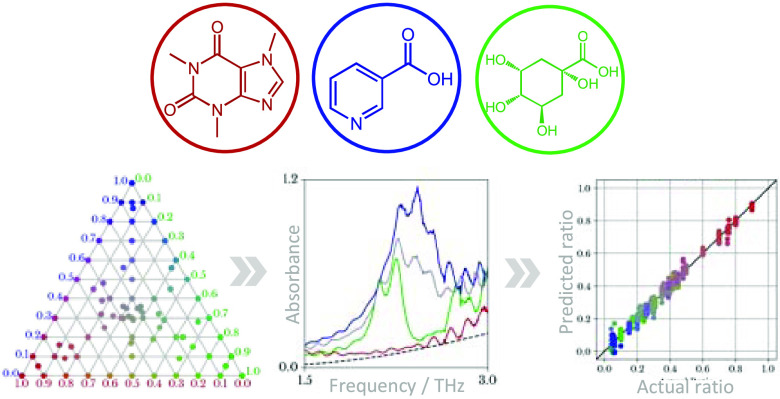

Caffeine, quinic acid, and nicotinic acid are among the significant chemical determinants of coffee quality. This study develops a chemometric model to quantify these compounds in ternary mixtures analyzed by terahertz time-domain spectroscopy (THz-TDS). A data set of 480 THz spectra was obtained from 80 samples. Combinations of data preprocessing methods, including normalization (*Z*-score, min-max scaling, Mie baseline removal) and dimensionality reduction (principal component analysis (PCA), factor analysis (FA), independent component analysis (ICA), locally linear embedding (LLE), non-negative matrix factorization (NMF), isomap), and prediction models (partial least-squares regression (PLSR), support vector regression (SVR), multilayer perceptron (MLP), convolutional neural network (CNN), gradient boosting) were analyzed for their prediction performance (totaling to 4,711,685 combinations). Results show that the highest quantification performance was achieved at a root-mean-square error of prediction (RMSEP) of 0.0254 (dimensionless mass ratio), using min-max scaling and factor analysis for data preprocessing and multilayer perceptron for prediction. Effects of preprocessing, comparison of prediction models, and linearity of data are discussed.

## 1 Introduction

**Figure 1:**
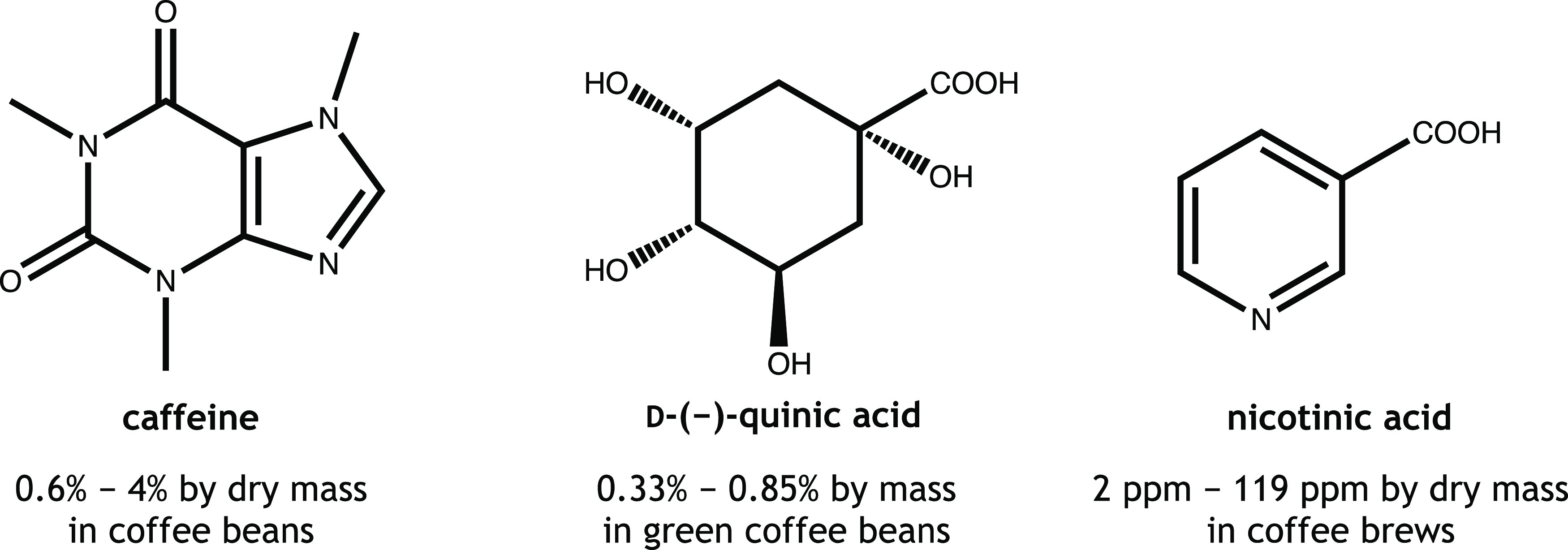
Chemical structure and content of caffeine, d-(−)-quinic acid, and nicotinic acid in coffee.

Coffee is one of the most widely consumed and traded luxury goods in the world. Coffee plant belongs to the genus *Coffea* in the family *Rubiaceae*. The most economically important species are *Coffea arabica L.*, or arabica coffee, and *Coffea canephora var. robusta* (L. Linden), or robusta coffee. Coffee is mostly produced commercially from either species or blends of both.^1^ Different blends of coffee with different degrees of roast vary by their body, mouthfeel, astringency, acidity, bitterness, flavors and aromas, and bioactive qualities.^2^ These qualities are attributed to the chemical composition of each cup of coffee. Caffeine, quinic acid, and nicotinic acid (Figure 1) are among the major molecular determinants in coffee. The content of these compounds in coffee can vary significantly in the literature, depending on cultivar, processing, and method of analysis.

Caffeine, or 1,3,7-trimethyl-xanthine, plays physical, chemical, and biological roles in determining the strength and body of coffee, influencing the bitterness of coffee, and being psychologically active, respectively.^3^ Caffeine can be found naturally in coffee beans, tea leaves, and cocoa beans and can be added to carbonated drinks, energy drinks, and other food and beverages.^4^ The caffeine content in coffee beans ranges from 0.6 to 4% by dry mass.^3^

Quinic acid is one of the determining factors for the taste, flavor and aroma, and acidity of coffee.^5^ As one of the major acids in coffee, it is found in green coffee beans and also derived from chlorogenic acids (CGAs) during the roasting process.^6^ The content of quinic acid ranges from 0.33 to 0.85% by mass in green coffee beans.^7^ Quinic acid content increases during the roasting process and is higher in darker roasted coffee due to a higher degree of CGA degradation. Quinic acid derivatives formed during the roasting process include bitter-tasting, flavor, and aroma compounds.^5^

Nicotinic acid (niacin; vitamin B3) is one of the micronutrients in coffee. It is a water-soluble B vitamin derived from trigonelline during coffee roasting. Coffee can be a significant dietary source of bioavailable nicotinic acid.^8^ Nicotinic acid content ranges from 2 to 119 ppm by dry mass in coffee brews.^9^ The amount of nicotinic acid produced varies with the degree of roasting.

Terahertz time-domain spectroscopy (THz-TDS) coupled with chemometric analysis has recently gained attention as a rapid, nondestructive method of qualitative and quantitative determination of compounds in food products. The terahertz regime is in the frequency range of 0.1–10 THz, corresponding to the wavelength range of 0.03–3 mm. This range is located between the microwave and infrared ranges. THz spectroscopy can reveal crucial molecular information of intramolecular and intermolecular modes caused by hydrogen bonds, van der Waals energy, torsional modes, and vibrational modes of molecules.^10^ THz waves have relatively low photon energy (4 meV for 1 THz; cf. 1.6–3.3 eV for visible light) and nonionizing penetration, causing minimal damage to biomolecules.^11^ In addition, THz waves are resistant to strong scattering^12^ and fluorescent substances^13^ in samples, which are issues arising in infrared and Raman spectroscopy, respectively. THz-TDS has been used with chemometric methods, from simple linear regression^14,15^ to machine learning models,^16−27^ to quantify nutritional, pharmaceutical, and explosive compounds. Applications of THz-TDS and chemometric methods in food analysis are summarized in Table 1.

**Table 1:** Recent Studies of THz-TDS Coupled with Chemometric Methods in Food Analysis

compound	sample matrix^a^	chemometric method^b^	reference
citric acid, d-(−)-fructose, d-(+)-lactose	n/a	PLSR, ANN	ref (16)
imidacloprid	rice powder	PLSR, SVR, iPLS, biPLS	ref (18)
l-(−)-glutamic acid, l-(−)-glutamine, l-(−)-tyrosine	cereal (foxtail millet)	PLSR, SVM	ref (20)
l-(−)-glutamic acid, l-(−)-glutamine, l-(−)-tyrosine	cereal (foxtail millet)	TM-stepwise regression, PLSR, *N*-PLSR	ref (21)
imidacloprid, carbendazim	flour	PLSR, PCA, SVM	ref (22)
d-(−)-fructose, d-(+)-galactose, d-(+)-mannose	n/a	PLSR, SVR	ref (23)
benzoic acid	flour	GRNN, BPNN	ref (24)
flavanoids	n/a	PLSR, ANN, PCA, SVM	ref (25)
proteins	soybean	PLSR, PCA-RBFNN, ABC-SVR	ref (26)
bisphenol A, bisphenol S, bisphenol AF, bisphenol E	n/a	SVR	ref (27)
caffeine, d-(−)-quinic acid, nicotinic acid	n/a	PLSR, SVR, MLP, CNN, gradient boost	this work

a Reported as n/a if pure samples with binder (polyethylene) are used.

b PLSR, partial least-squares regression; ANN, artificial neural networks; SVR, support vector regression; iPLS, interval partial least squares; biPLS, backward interval partial least squares; SVM, support vector machine; TM, Tchebichef image moment; *N*-PLSR, *N*-way partial least-squares regression; PCA, principal component analysis; GRNN, generalized regression neural network; BPNN, back-propagation neural network; RBFNN, radial basis function neural network; ABC, artificial bee colony; MLP, multilayer perceptron; and CNN, convolutional neural network.

In this study, ternary mixtures of caffeine, quinic acid, and nicotinic acid are analyzed by THz-TDS and a chemometric model for the quantification of these compounds is developed. Combinations of preprocessing techniques and prediction models are assessed for their quantification performance.

## 2 Results and Discussion

**Figure 2:**
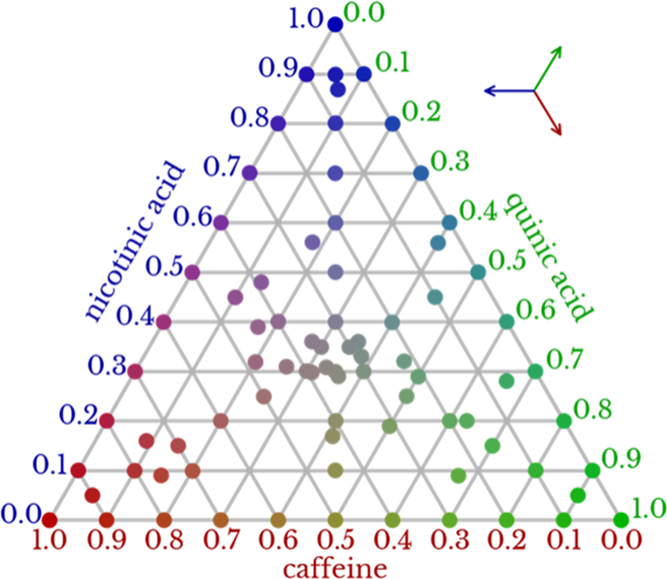
Ternary diagram of 80 sample compositions analyzed by THz-TDS. To read the mixture compositions (dimensionless mass ratio), the key on the top right can be placed on a point, extending the lines to the axes or triangle edges. Preparation methods are described in Section 4.1.

A series of 480 measurements of 80 samples with different compositions were analyzed by THz-TDS. Sample compositions were systematically and randomly determined, as shown in Figure 2. Replicate spectral measurements were conducted at different points on the samples. Further details can be found in Section 4.1.

**Figure 3:**
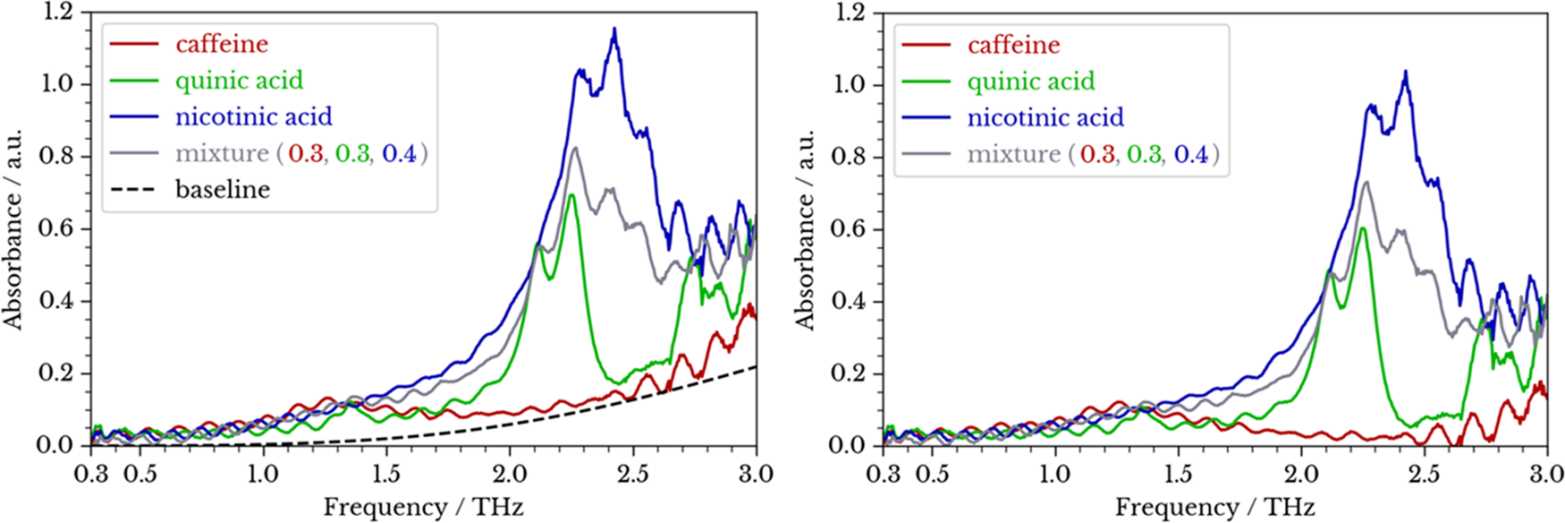
THz absorption spectra of pure caffeine, quinic acid, nicotinic acid, and a ternary mixture before (left) and after (right) Mie baseline removal (a.u. = arbitrary unit).

Examples of the obtained absorbance spectra are shown in Figure 3. The THz spectra of pure compounds have been explored using experimental and computational methods and reported in the literature. The absorption peaks observed in Figure 3, especially those in the 2.0–3.0 THz range, are in good agreement with previous THz-TDS studies on caffeine,^28^ quinic acid,^29^ and nicotinic acid.^30^

The *k*-fold cross-validation method was used to evaluate the performance of each chemometric method. Spectra of ternary mixtures (*n* = 300) were divided into five parts (each with *n* = 60), whereby one part is in the test set and the rest are in the training set of each fold (e.g., part one is the test set of fold one, part two is the test set of fold two, etc.). Spectra of unitary and binary mixtures (*n* = 180) were always in the training sets. This is to resemble the use of the model in practice, whereby unitary and binary mixtures set boundaries (or extrema) for the (potentially nonlinear) interpolation of ternary mixtures. Each model was assessed using root-mean-square error of calibration (RMSEC), RMSE for the training set, and root-mean-square error of prediction (RMSEP), RMSE for the test set.

**Figure 4:**
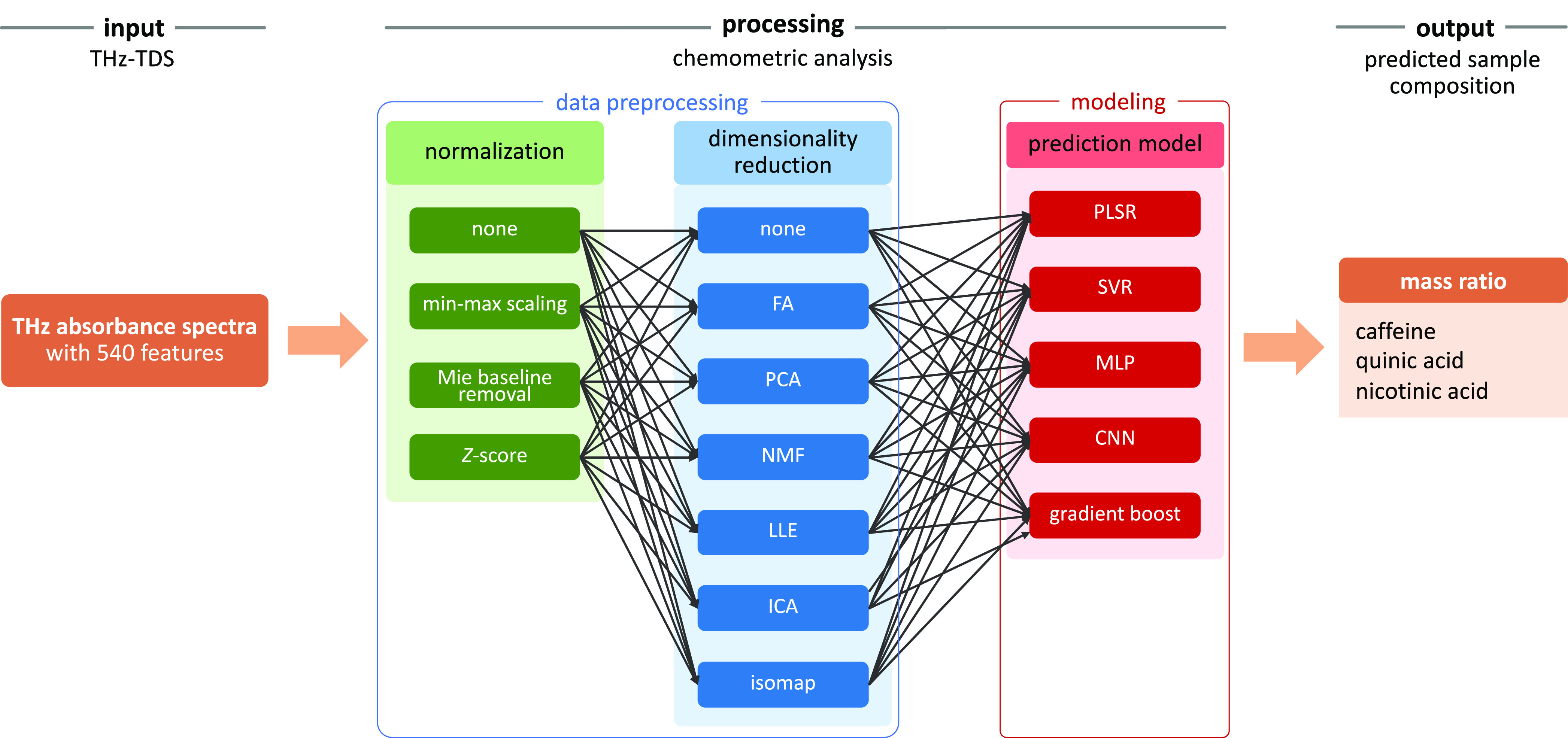
Pipeline of chemometric methods. See the list of abbreviations in Table 4.

Multiple chemometric models were examined in this study, as shown in Figure 4. Absorbance spectra of 540 features or data points were used as high-dimensional inputs for the models. Two data preprocessing measures were explored to understand their effect on the performance of the models. Normalization is performed to ensure faster convergence, and dimensionality reduction techniques were performed to remove noises and only retain meaningful features. Each preprocessing step (normalization and dimensionality reduction) provides a bypass (no preprocessing) configuration, so each prediction model can have (1) both normalization and dimensionality reduction, (2) only normalization, (3) only dimensionality reduction, and (4) no preprocessing. Three normalization techniques, six dimensionality reduction techniques, and five prediction models were investigated in this study (see details in Section 4.2).

**Figure 5:**
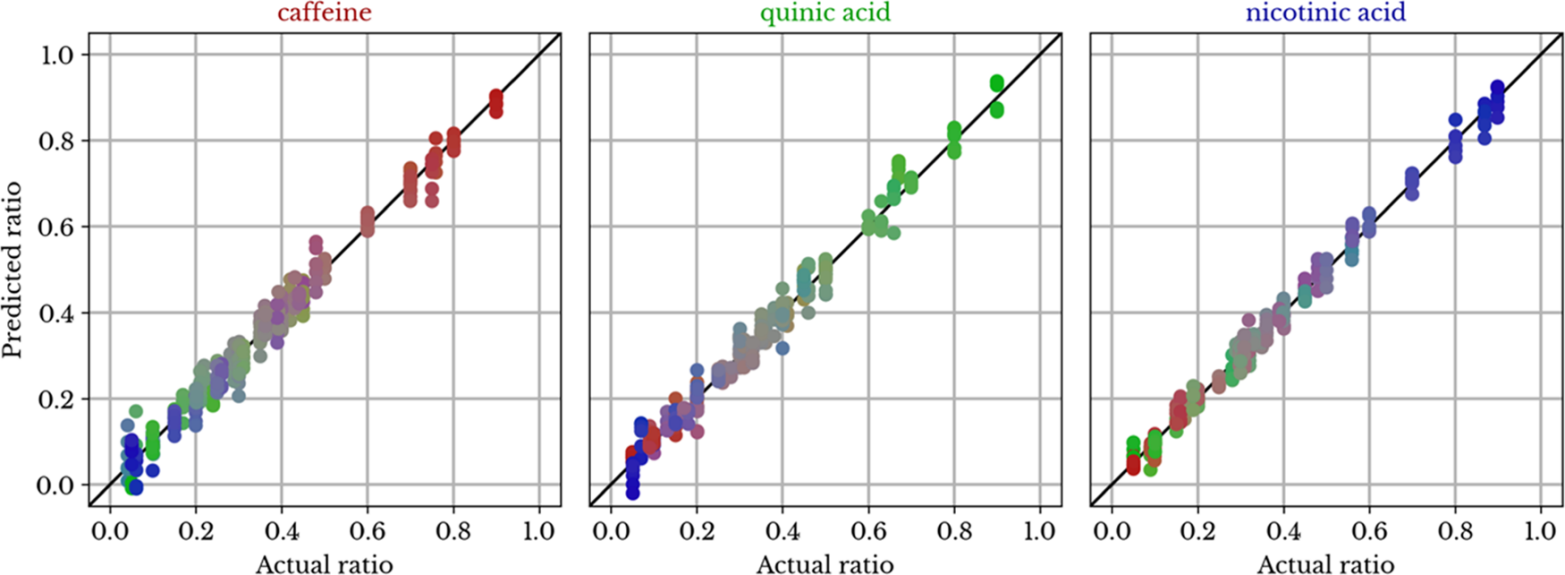
Performance of MLP with min-max scaling and FA preprocessing: predicted vs actual (dimensionless) mass ratio of caffeine (red), quinic acid (green), and nicotinic acid (blue) in ternary mixtures (point colors are proportional to their associated predicted composition—see Figure 2).

A list of chemometric models (combinations of preprocessing techniques and prediction models) with their respective RMSE values can be found in the Supporting Information. Preprocessing techniques resulting in the lowest RMSEP in each prediction model are shown in Table 2. Note that the best-performing PLSR (linear model) utilizes a nonlinear dimensionality reduction method. The nonlinear effect is further explained in Section 2.3. The lowest RMSEP of 0.0254 was achieved with the combination of min-max scaling, FA, and MLP prediction. The prediction performance of this model is visualized in Figure 5. The physical interpretation of parameters is not explicit in most of the investigated models due to the multiple layers of processing. A spectral feature may seem significant in a layer, yet be discarded in subsequent layers.

**Table 2:** Highest-Performance Preprocessing Technique by RMSEP for Each Prediction Model and Their Respective Optimal Hyperparameters^a^

model	normalization	dimensionality reduction	RMSEC	RMSEP
MLP	min-max scaling	FA	0.0213	0.0254
#neurons: 4, activation fn: logistic, solver: lbfgs		27 components		
SVR	Mie baseline removal	PCA	0.0262	0.0260
type: NuSVR, nu: 0.5, C: 1.0, iterations: 2000, kernel: rbf, γ: auto	multifactor	27 components		
CNN	Mie baseline removal	none	0.0276	0.0293
activation fn: sigmoid	multifactor (with factor concatenation)			
gradient boosting	Mie baseline removal	none	0.0214	0.0316
learning rate: 0.01, max depth: 5, min child weight: 2, γ: 0, subsample: 0.3, colsample_bytree: 0.6, num_round: 10,000	multifactor			
PLSR	Mie baseline removal	LLE	0.0312	0.0283
with prescale, 3 components	multifactor	modified with 14 components, 30 neighbors		

a Abbreviations are listed in Table 4. RMSEC and RMSEP values are reported as dimensionless mass ratios.

### 2.1 Effect of Preprocessing

Data preprocessing measures are performed to improve the performance of prediction models. However, these techniques might not always improve the prediction performance. In some instances, they can remove useful features and correlations that are beneficial to the prediction model. As shown in Table 2, the CNN and gradient boosting models performed best without dimensionality reduction. The best CNN prediction was achieved when coupled with Mie baseline removal, with an RMSEP value of 0.0293. The CNN model without data preprocessing, on the other hand, resulted in a slightly higher RMSEP value of 0.0302. This shows how prediction models like CNN may already be able to extract useful features and data correlations without the need for data preprocessing, especially when the sample size is sufficient.

The effects of different data preprocessing methods on prediction performance are examined. The RMSEPs of models with one or more preprocessing techniques (normalization and/or dimensionality reduction) were compared to the corresponding combination with one of the techniques not applied. For example, to examine the effect of the min-max scaling normalization technique, the RMSEP of each model with no normalization is subtracted from the RMSEP of the model with the min-max scaling and the same dimensionality reduction and prediction models. A negative value of this difference signifies a reduction in RMSEP, hence an improvement in the prediction performance. Mean differences for each model are reported in Table 3. RMSE values of models with no preprocessing and plots of differences can be found in the Supporting Information.

**Table 3:** Mean Change in RMSEP for Each Prediction Model by Different Preprocessing Methods^a^

		mean change in RMSEP				
preprocessing technique		PLSR	SVR	MLP	CNN	gradient boost
normalization	Mie baseline removal	–0.3326	–0.1252	–0.0063	–0.0087	–0.0071
	min-max scaling	–0.0866	0.3918	0.0088	0.0144	0.0130
	*Z*-score	0.0732	1.0256	0.0282	0.0273	0.0240
dimensionality reduction	FA	–0.0047	–0.3354	–0.0109	0.0249	0.0055
	ICA	–0.0037	–0.3356	0.0312	0.0443	0.0114
	isomap	0.0229	0.6478	0.0108	0.0316	0.0239
	LLE	–0.0021	–0.4042	0.0305	0.0080	0.0097
	NMF	0.0001	–0.4179	0.0118	0.0129	0.0080
	PCA	–0.0026	0.9325	–0.0050	0.0133	0.0119

a RMSEP values are reported as dimensionless mass ratios.

PLSR with PCA preprocessing is among the most widely used chemometric model. In this study, implementing PCA preprocessing with PLSR led to a mean RMSEP reduction of 0.0026, compared to the PLSR model with no dimensionality reduction. This improvement can be attributed to how PCA maximizes the covariance among the input data, while PLSR maximizes the covariance between the input and output. PCA can be perceived as a filtering layer that discards noise, which can aid the covariance maximization of PLSR. Nevertheless, other nonlinear dimensionality reduction techniques like FA and ICA resulted in greater RMSEP reduction for PLSR models. Therefore, it can be beneficial to explore less common preprocessing techniques to improve the performance of prediction models. For normalization techniques, Mie baseline removal performs relatively well on average for all models.

Data preprocessing techniques resulted in reductions as well as increases in RMSEP. The increases can be attributed to how, in some instances, preprocessing can discard useful features that compromise the performance of prediction models. It is important to select the right combinations of preprocessing techniques and prediction models to enhance the effectiveness of each process and optimize the prediction performance.

### 2.2 Prediction Model

The combinations of data preprocessing methods resulting in the best prediction performance for each model are shown in Table 2. PLSR is a widely used machine learning model, and PCA is among the most common dimensionality reduction methods used with it. According to the results, Mie baseline removal and LLE preprocessing resulted in the best-performing PLSR prediction with an RMSEP of 0.0282, while Mie baseline removal and PCA preprocessing resulted in a higher RMSEP of 0.0292. Thus, according to this study, PLSR prediction can be optimized by data preprocessing of Mie baseline removal and LLE modified with 14 components and 30 neighbors.

### 2.3 Linearity of Data

**Figure 6:**
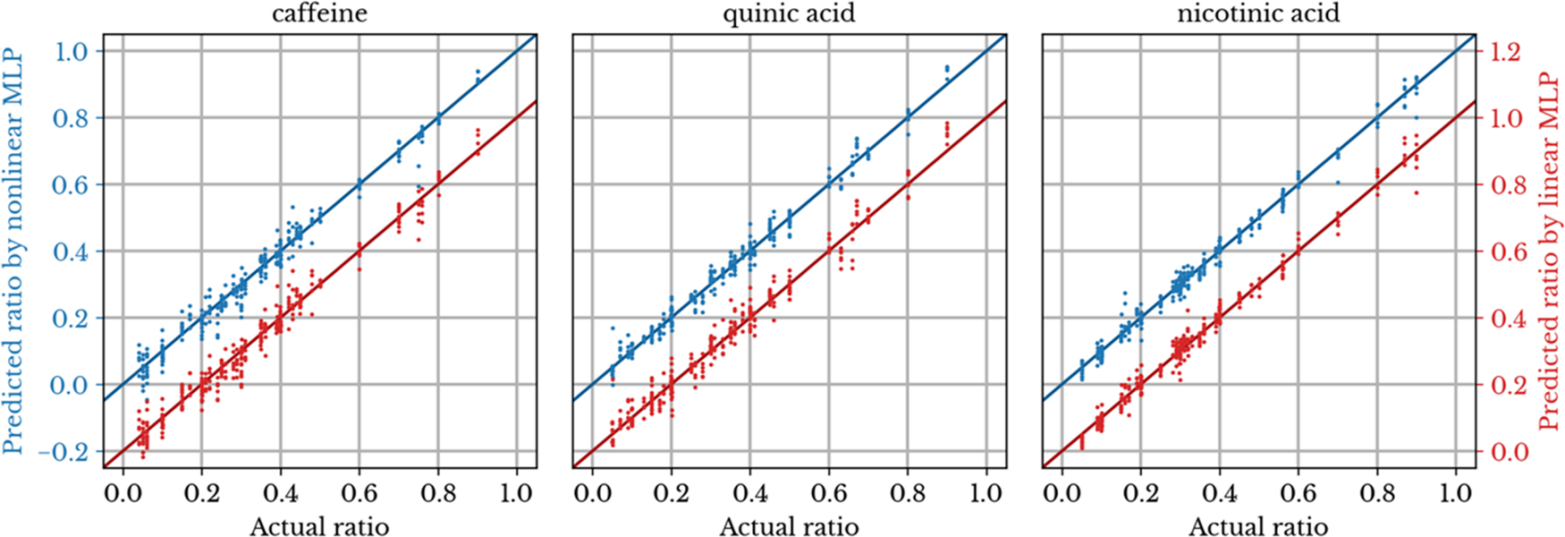
Prediction performance of nonlinear (blue) and linear (red) MLP models for (dimensionless) mass ratios of caffeine, quinic acid, and nicotinic acid in ternary mixtures. The left vertical axis is shifted up with respect to the right vertical axis for ease of comparison.

The linearity between the input (spectral data) and output (mixture composition) was investigated. This was done by modifying the activation function in the hidden layer and output layer (“tanh” function for nonlinear model and “identity” function for linear model) of the MLP model. Linear and nonlinear MLP models with no preprocessing are compared. The best nonlinear MLP model has a 14% lower RMSEP compared to the linear model (0.0270 vs 0.0316). The prediction performances of these models are visualized in Figure 6. The MLP nonlinear models resulted in lower RMSEP values than those of the linear models in most cases. (see the Supporting Information). This suggests a nonlinear relationship between our input data and mixture composition.

## 3 Conclusions

In this study, ternary mixtures of caffeine, quinic acid, and nicotinic acid with varying compositions were analyzed by THz-TDS, and chemometric models were investigated for their performance in the prediction of mixture compositions. The performance of different prediction models, as well as the effect of different data preprocessing techniques on the performance, was explored. Among all models, MLP with the preprocessing techniques of min-max scaling and factor analysis (with 27 components) had the best prediction performance with a dimensionless mass ratio RMSEP of 0.0254. The widely used PLSR prediction model achieved its best prediction results when Mie baseline removal and LLE dimensionality reduction were performed. In addition, a nonlinear model resulted in better prediction performance than the linear model, using MLP as a model method.

The developed chemometric models can serve as a stepping stone for further investigation in food, pharmaceutical, and cosmetic products containing these compounds, such as coffee, energy drinks, and supplements. Additional practical considerations should be taken into account when applying the techniques to the products.

## 4 Materials and Methods

### 4.1 THz-TDS Spectroscopy

All chemicals used are analytical grade (>98% purity) and in their anhydrous forms. Caffeine was purchased from LobaChemie; d-(−)-quinic acid and nicotinic acid were obtained from Sigma-Aldrich. A high-density polyethylene (HDPE) powder purchased from Sigma-Aldrich was used as the binder for all sample mixtures. All chemicals were used as received without any further purification.

The mixture compositions shown in Figure 2 include 60 and 20 systematically and randomly determined compositions, respectively. The systematically determined compositions include 10 points evenly spaced on each of the three edges and three medians of the ternary diagram (triangle). Each sample was mixed with HDPE at a 20:80 mass ratio and ground to avoid aggregates and heterogeneous clusters. Each mixture was transferred to a hydraulic press to be formed into disc pellets at 7 tons for 5 min. Each disc pellet had an approximately 100 mg of weight, 13 mm of diameter, and 0.850–0.950 mm of thickness. In addition to the samples, pure HDPE was prepared to be used as a blank (reference).

Six spectral measurements of each composition were performed for a total of 480 spectra. Each measurement was performed at different points on the samples. Four measurement points were arranged in a square shape, and two measurement points (the first and the last) were placed in the center of the square, 1.0 mm apart from the other four.

**Figure 7:**
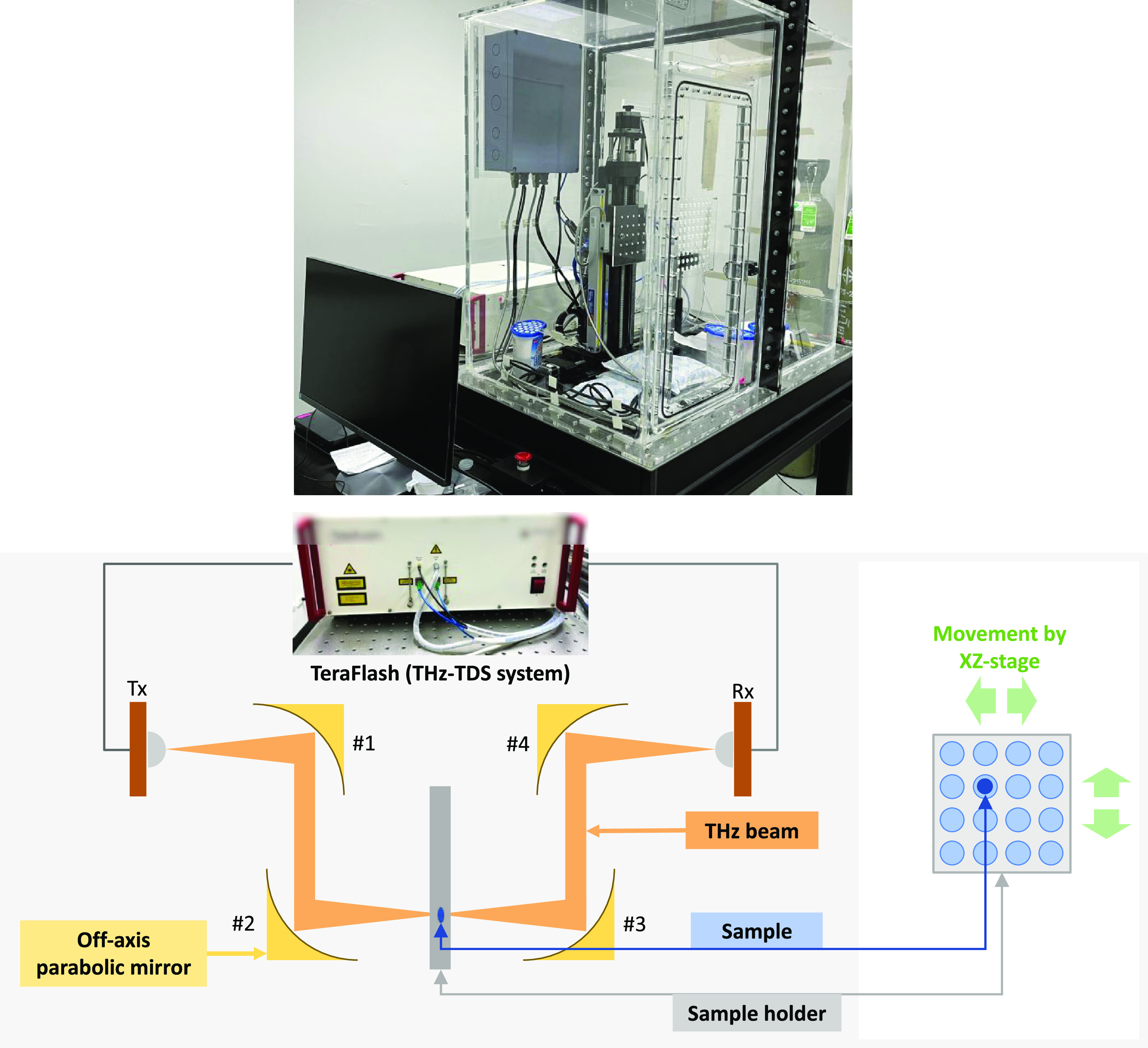
Custom-made THz-TDS system at NECTEC, Pathum Thani, Thailand. The photoconductive antenna transmitter and receiver are labeled as Tx and Rx, respectively. Off-axis parabolic mirrors are labeled as #1, #2, #3, and #4.

Absorption spectra of the range 0.3–3.0 THz were obtained from a THz-TDS system (TeraFlash, TOPTICA Photonics AG, Germany), as shown in Figure 7. The source of radiation is a photoconductive switch generated by a femtosecond laser at 780 nm. The experiment was conducted at an air-conditioned room temperature (25 ± 2 °C). The acrylic chamber with a nitrogen-purged atmosphere (relative humidity ≤5%) was custom-made by researchers at the National Electronics and Computer Technology Center (NECTEC), Thailand. The spectra were acquired at a 5 GHz resolution, optimized, and formed a time-domain data set {*x*_*i*_, *y*_*i*_ | *x*_*i*_ ∈ **R**^4001^, *y*_*i*_ ∈ **R**^3^, *i* = 1, 2, ···, *n*} of *n* samples, where each sample contained vector *x*_*i*_ and target concentration *y*_*i*_. Each time-domain spectrum was obtained from a 2000-sample moving average. For background compensation, frequency-domain data were subtracted from the reference spectra.

### 4.2 Chemometric Models

Data preprocessing methods of normalization (three techniques) and dimensionality reduction (six techniques) and five prediction models were explored in this study, as described in Table 4. An exhaustive investigation of hyperparameters for each preprocessing technique and prediction model was conducted to identify the optimal hyperparameters reported in Table 2. A total of 4,711,685 combinations of preprocessing techniques and models were explored. Additionally, a novel Mie baseline removal method in the form of convex optimization is proposed and used in this study, as described in Table 4.

**Table 4:** List of Investigated Chemometric Techniques with Brief Description

process	technique	description
normalization	*Z*-score	features are transformed so that the mean is 0 and the standard deviation is 1.
	min-max scaling	features are transformed so that data is in the range of [0, 1].
	Mie baseline removal	Mie scattering caused by the interaction of the HDPE binder with THz waves is corrected.
		Mie efficiency (*Q*_ext_) for multiple wavelengths was generated by Matzler.^34^ In this study, the baseline is calculated as ξ·*Q*_ext_, where ξ is a coefficient computed such that the baseline is at the troughs.
		we regarded this as a convex optimization problem to a valid set of discretized angular frequencies Ω, which excludes the water peaks (with ±25 GHz bandwidth) and the frequencies ranging from 0.3 to 0.55 THz due to negative absorbance residues arising from noises. We denoted absorbance as *a*(ω) and baseline as ξ·*Q*_ext_(ω). The optimization target was to minimize the following.

		ξ is the solution found after optimization. *M* is an arbitrarily large number to keep the baseline from exceeding the spectra troughs.
		◦ for single-factor, ξ is a constant scalar value. Minimum ξ computed in a training set is used among all spectra, including the test set.
		◦ for multifactor, ξ can have different values, and the baseline of each spectrum is individually removed. A special case of multifactor concatenates the ξ parameters after dimensionality reductor as another feature.
dimensionality reduction	principal component analysis (PCA)	data is projected onto a low-dimensional linear space formed by principal orthogonal components.
		cumulative variance in the predictors is explained.
	factor analysis (FA)	data is projected onto a low-dimensional linear space formed by factors that correlate with other variables.
		correlations between variables are explained.
	independent component analysis (ICA)	data is projected onto a low-dimensional linear space formed by maximally independent components.
	locally linear embedding (LLE)	data is projected onto a low-dimensional nonlinear space, preserving distances between neighboring points.
	non-negative matrix factorization (NMF)	data is projected onto a low-dimensional linear space formed by non-negative additive factors.
	isomap	data is projected onto a low-dimensional nonlinear space, preserving geodesic distances.
prediction model	partial least-squares regression (PLSR)	least-squares regression is performed on dimensionally reduced input data with the retained correlation between data.
	support vector regression (SVR)	hyperplane parameters, whose margin of error confines as many training data points as possible, are obtained and used for prediction. Outliers tend to be far away from the margin of error.
	multilayer perceptron (MLP)	nonlinear function approximator is obtained for either classification or regression, and parameters are adjusted using “back-propagation”. Hidden and output layers contain neurons using an activation function. Data is fed from one layer to another.
	convolutional neural network (CNN)	nonlinear mapping function (residual block^24^) between the input and output is obtained. Data features are extracted through the convolution of input and filters.
	gradient boosting	model is developed from a combination of trained base learners. XGBoost is an optimized distributed gradient boosting library that has regularization parameters to prevent overfitting and was selected in this study.

Source codes were implemented using Python and the scikit-learn,^31^ Keras,^32^ and XGBoost^33^ Python libraries. Jupyter notebook was used as the integrated development environment. Optimization of hyperparameters was performed on ray.io. All data and source codes are available in the Supporting Information. The total runtime for our computational investigation described in the paper is approximately 3 days on AWS Graviton 2 (256 cores, 1 TB RAM). However, an individual model can be computed locally on a standard workstation (4 cores, 8 GB RAM) in less than 1 hour.

## Notes

The prediction performance of all of the investigated models can be accessed via Open Science Framework (OSF), at DOI: 10.17605/OSF.IO/3YXBU.

## Supporting Information Available

Distribution of change in the RMSE of each prediction model using different normalization techniques and dimensionality reduction techniques for preprocessing, and highest-performance RMSEP for each prediction model with no preprocessing and their respective optimal hyperparameters (ZIP)
Raw time-domain and processed frequency-domain spectral data and source codes in Jupyter notebooks for data extraction and analysis, including a test case from the best performing models (ZIP)
