# Supplementary material for: Chemometric Analysis of a Ternary Mixture of Caffeine, Quinic Acid, and Nicotinic Acid by Terahertz Spectroscopy
Source: ACS Omega. 2022 Sep 27;7(40):35783–91. doi: 10.1021/acsomega.2c03808 (PMC9558605; doi:10.1021/acsomega.2c03808)
Supplement: Supplementary file 1 — ao2c03808_si_001.zip [file ao2c03808_si_001.zip › index.docx]

**Supporting Information**

**Chemometric Analysis of Ternary Mixture of Caffeine, Quinic Acid and Nicotinic Acid by Terahertz Spectroscopy**

Phatham Loahavilai^a,b^, Sopanant Datta^c^, Kiattiwut Prasertsuk^a^, Rungroj Jintamethasawat^a^, Patharakorn Rattanawan^a^, Jia Yi Chia^a^, Cherdsak Kingkan^a^, Chayut Thanapirom^a^ and Taweetham Limpanuparb^c,*^

^a^National Electronics and Computer Technology Center, 112 Thailand Science Park, Pathum Thani, 12120, Thailand
^b^Department of Engineering Physics, Tsinghua University, Beijing, 100084, China
^c^Mahidol University International College, Mahidol University, Salaya, Nakhon Pathom, 73170, Thailand

**Items in this document**

- Figure S1: Distribution of change in RMSE for each prediction model by using different normalization techniques for preprocessing
- Figure S2: Distribution of change in RMSE for each prediction model by using different dimensionality reduction techniques for preprocessing
- Table S1: Highest–performance RMSEP for each prediction model with no preprocessing and their respective optimal hyperparameters

**Items in data_and_codes folder**

- raw time-domain and processed frequency-domain spectral data
- source codes in Jupyter notebooks used for data extraction and analysis

**Items in DOI: 10.17605/OSF.IO/3YXBU (accessed via osf.io)**

- prediction performance (RMSE) of all investigated models
- actual and predicted mass ratio of all samples


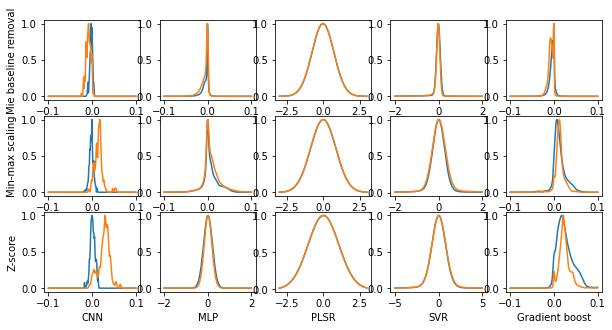


**Figure S1** Distribution of change in RMSEC (blue) and RMSEP (orange) for each prediction model by using different normalization techniques for preprocessing


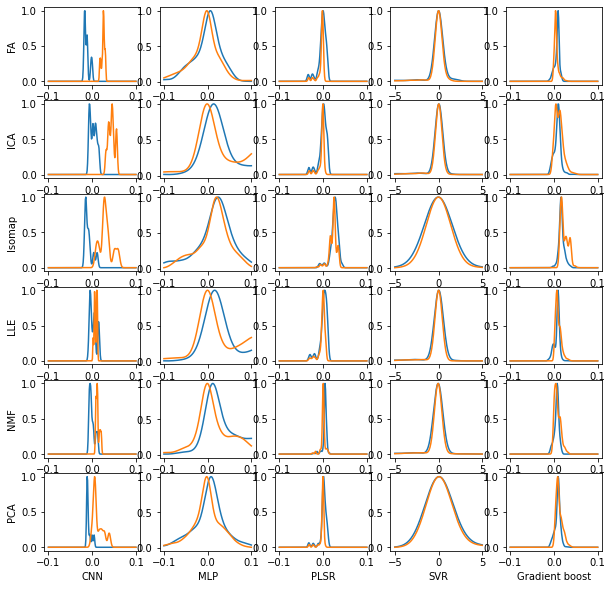
**Figure S2** Distribution of change in RMSEC (blue) and RMSEP (orange) for each prediction model by using different dimensionality reduction techniques for preprocessing

**Table S1** Highest–performance Prediction Model (with No Preprocessing) by RMSEP and Their Respective Optimal Hyperparameters

| **model** | **normalization** | **dimensionality reduction** | **RMSEC** | **RMSEP** |
| --- | --- | --- | --- | --- |
| MLP  # neurons: 16,  activation fn: tanh,  solver: lbfgs | none | none | 0.0154 | 0.0270 |
| SVR  type: NuSVR, nu: 0.7, C: 1.0,  iterations: 2000, kernel: rbf,  gamma: scale | none | none | 0.0241 | 0.0279 |
| CNN  activation fn: sigmoid | none | none | 0.0331 | 0.0302 |
| Gradient boosting  learning rate: 0.01,  max depth: 5,  min child weight: 2,  gamma: 0, subsample: 0.4  colsample_bytree: 0.6,  num_round: 10000 | none | none | 0.0231 | 0.0393 |
| PLSR  without prescale,  12 components | none | none | 0.0291 | 0.0296 |
