# Supplementary material for: Chemometric Analysis of a Ternary Mixture of Caffeine, Quinic Acid, and Nicotinic Acid by Terahertz Spectroscopy
Source: ACS Omega. 2022 Sep 27;7(40):35783–91. doi: 10.1021/acsomega.2c03808 (PMC9558605; doi:10.1021/acsomega.2c03808)
Supplement: Supplementary file 1 — ao2c03808_si_001.zip [file ao2c03808_si_001.zip › index.pdf]

Supporting Information

Chemometric Analysis of Ternary Mixture of Caffeine, Quinic Acid and Nicotinic Acid by Terahertz Spectroscopy

Phatham Loahavilai[a,b], Sopanant Datta[c], Kiattiwut Prasertsuk[a], Rungroj Jintamethasawat[a], Patharakorn Rattanawan[a], Jia Yi Chia[a], Cherdsak Kingkan[a], Chayut Thanapirom[a] and Taweetham Limpanuparb[c,*]

[a]National Electronics and Computer Technology Center, 112 Thailand Science Park, Pathum Thani, 12120, Thailand
[b]Department of Engineering Physics, Tsinghua University, Beijing, 100084, China
[c]Mahidol University International College, Mahidol University, Salaya, Nakhon Pathom, 73170, Thailand

Items in this document

- Figure S1: Distribution of change in RMSE for each prediction model by using different normalization techniques for preprocessing
- Figure S2: Distribution of change in RMSE for each prediction model by using different dimensionality reduction techniques for preprocessing
- Table S1: Highest–performance RMSEP for each prediction model with no preprocessing and their respective optimal hyperparameters

Items in data_and_codes folder

- raw time-domain and processed frequency-domain spectral data
- source codes in Jupyter notebooks used for data extraction and analysis

Items in DOI: 10.17605/OSF.IO/3YXBU (accessed via osf.io)

- prediction performance (RMSE) of all investigated models
- actual and predicted mass ratio of all samples

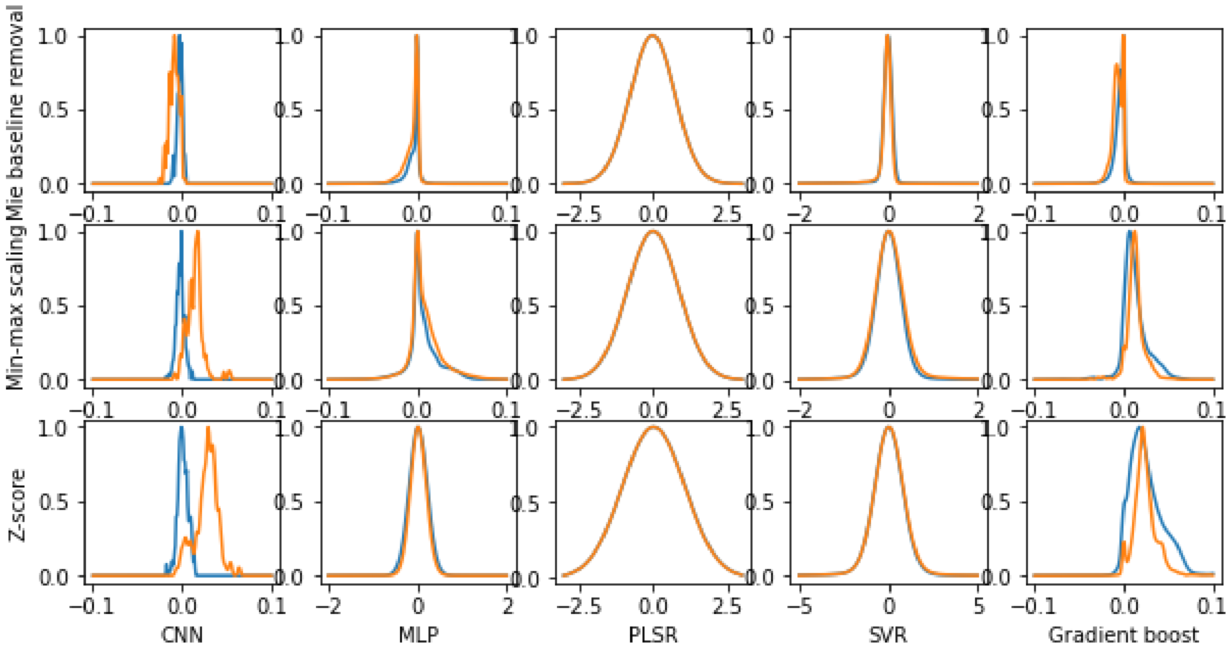

Figure S1 Distribution of change in RMSEC (blue) and RMSEP (orange) for each prediction model by using different normalization techniques for preprocessing

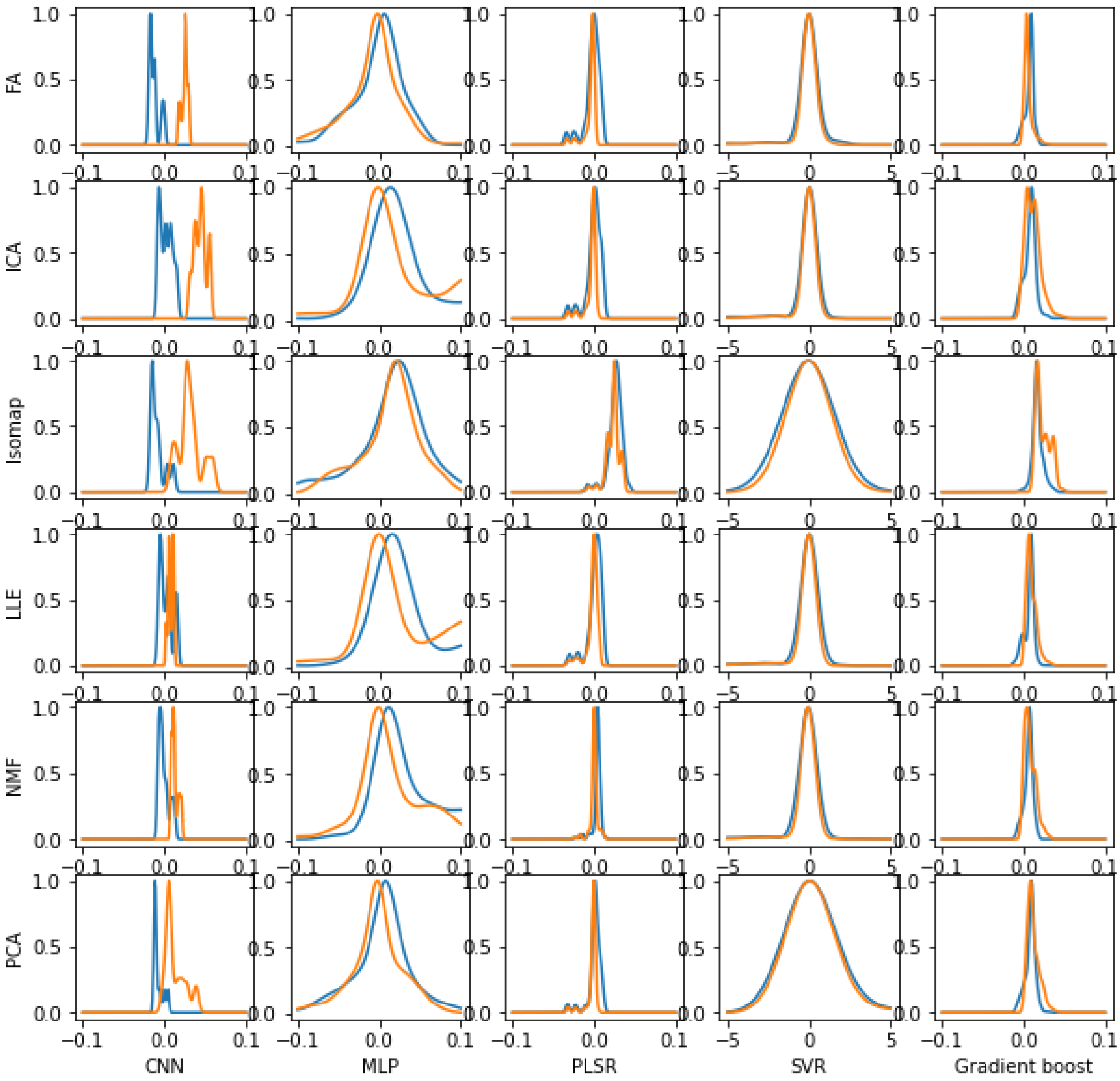

Figure S2 Distribution of change in RMSEC (blue) and RMSEP (orange) for each prediction model by using different dimensionality reduction techniques for preprocessing

Table S1 Highest–performance Prediction Model (with No Preprocessing) by RMSEP and Their Respective Optimal Hyperparameters

model	normalization	dimensionality reduction	RMSEC	RMSEP
MLP<br># neurons: 16,<br>activation fn: tanh,<br>solver: lbfgs	none	none	0.0154	0.0270
SVR<br>type: NuSVR, nu: 0.7, C: 1.0,<br>iterations: 2000, kernel: rbf,<br>gamma: scale	none	none	0.0241	0.0279
CNN<br>activation fn: sigmoid	none	none	0.0331	0.0302
Gradient boosting<br>learning rate: 0.01,<br>max depth: 5,<br>min child weight: 2,<br>gamma: 0, subsample: 0.4<br>colsample_bytree: 0.6,<br>num_round: 10000	none	none	0.0231	0.0393
PLSR<br>without prescale,<br>12 components	none	none	0.0291	0.0296
